# Assessment of Trends in Guideline-Based Oral Anticoagulant Prescription for Patients With Ischemic Stroke and Atrial Fibrillation in China

**DOI:** 10.1001/jamanetworkopen.2021.18816

**Published:** 2021-07-29

**Authors:** Hong-Qiu Gu, Xin Yang, Chun-Juan Wang, Xing-Quan Zhao, Yi-Long Wang, Li-Ping Liu, Xia Meng, Yong Jiang, Hao Li, Chelsea Liu, Yun-Yun Xiong, Gregg C. Fonarow, David Wang, Ying Xian, Zi-Xiao Li, Yong-Jun Wang

**Affiliations:** 1 China National Clinical Research Center for Neurological Diseases, Beijing Tiantan Hospital, Capital Medical University, Beijing, China; 2 National Center for Healthcare Quality Management in Neurological Diseases, Beijing Tiantan Hospital, Capital Medical University, Beijing, China; 3 Vascular Neurology, Department of Neurology, Beijing Tiantan Hospital, Capital Medical University, Beijing, China; 4 Neuro-intensive Care Unit, Department of Neurology, Beijing Tiantan Hospital, Capital Medical University, Beijing, China; 5 Department of Epidemiology, Harvard T.H. Chan School of Public Health, Boston, Massachusetts; 6 Ahmanson-UCLA Cardiomyopathy Center, Ronald Reagan-UCLA Medical Center, Los Angeles, California; 7 Neurovascular Division, Department of Neurology, Barrow Neurological Institute, St Joseph’s Hospital and Medical Center, Phoenix, Arizona; 8 Department of Neurology, Duke University Medical Center, Durham, North Carolina; 9 Chinese Institute for Brain Research, Beijing, China

## Abstract

**Importance:**

Adherence to oral anticoagulants (OACs) per guideline recommendations is crucial in reducing ischemic stroke and systemic thromboembolism in high-risk patients with ischemic stroke and atrial fibrillation. However, data on OAC use are underreported in China.

**Objective:**

To assess adherence to the Chinese Stroke Association or the American Heart Association/American Stroke Association’s clinical management guideline–recommended prescription of OACs, the temporal improvement in adherence, and the risk factors associated with OAC prescriptions.

**Design, Setting, and Participants:**

This quality improvement study was conducted at 1430 participating hospitals in the Chinese Stroke Center Alliance (CSCA) among patients with ischemic stroke and atrial fibrillation enrolled in the CSCA between August 1, 2015, and July 31, 2019.

**Exposure:**

Calendar year.

**Main Outcomes and Measures:**

Adherence to the Chinese Stroke Association or the American Heart Association/American Stroke Association’s clinical management guideline–recommended prescribing of OACs (warfarin and non–vitamin K OACs, including dabigatran, rivaroxaban, apixaban, and edoxaban) at discharge.

**Results:**

Among 35 767 patients (18 785 women [52.5%]; mean [SD] age, 75.5 [9.2] years) with previous atrial fibrillation at admission, the median CHA_2_DS_2_-VASc (cardiac failure or dysfunction, hypertension, age 65-74 [1 point] or ≥75 years [2 points], diabetes, and stroke, transient ischemic attack or thromboembolism [2 points]–vascular disease, and sex category [female]) score was 4.0 (interquartile range, 3.0-5.0); 6303 (17.6%) were taking OACs prior to hospitalization for stroke, a rate that increased from 14.3% (20 of 140) in the third quarter of 2015 to 21.1% (118 of 560) in the third quarter of 2019 (*P*_ _< .001 for trend). Of 49 531 eligible patients (26 028 men [52.5%]; mean [SD] age, 73.4 [10.4] years), 20 390 (41.2%) had an OAC prescription at discharge, an increase from 23.2% (36 of 155) in the third quarter of 2015 to 47.1% (403 of 856) in the third quarter of 2019 (*P* < .001 for trend). Warfarin was the most commonly prescribed OAC (11 956 [24.2%]) and had the largest temporal increase (from 5.8% [9 of 155] to 20.7% [177 of 856]). Older age (adjusted odds ratio [aOR] per 5 year increase, 0.89;95% CI, 0.89-0.90), lower levels of education (aOR for below elementary school, 0.84; 95% CI, 0.74-0.95 ), lower income (aOR for ≤¥1000 [$154], 0.66; 95% CI, 0.59-0.73), having new rural cooperative medical scheme insurance (aOR, 0.92; 95% CI, 0.87-0.96), prior antiplatelet use (aOR, 0.70; 95% CI, 0.66-0.74), having several cardiovascular comorbid conditions (including stroke or transient ischemic attack [aOR, 0.78; 95% CI, 0.75-0.82], hypertension [aOR, 0.84; 95% CI, 0.80-0.89], diabetes [aOR, 0.91; 95% CI, 0.83-0.99], dyslipidemia [aOR, 0.87; 95% CI, 0.80-0.94], carotid stenosis [aOR, 0.83; 95% CI, 0.69-0.98], and peripheral vascular disease [aOR, 0.80; 95% CI, 0.71-0.90]), and admission to secondary hospitals (aOR, 0.71; 95% CI, 0.68-0.74) or hospitals located in the central region of China (aOR, 0.80; 95% CI, 0.75–0.84) were associated with not being prescribed an OAC at discharge.

**Conclusions and Relevance:**

This quality improvement study suggests that, despite significant improvement over time, OAC prescriptions remained low. Efforts to increase OAC prescriptions, especially non–vitamin K OACs, are needed for vulnerable subgroups by age, socioeconomic status, and presence of comorbid conditions.

## Key Points

**Question:**

Do prescribers in China follow established guidelines for prescription of oral anticoagulants (OACs) to patients with ischemic stroke and atrial fibrillation, and has adherence to prescribing guidelines changed over time?

**Findings:**

In this multicenter quality improvement study, among 35 767 eligible patients with ischemic stroke and atrial fibrillation at admission, fewer than 1 in 5 were taking OACs at admission, and of 49 531 eligible patients at discharge, only 41% were prescribed OACs at discharge. Although adherence to OACs has significantly improved over time, it remains suboptimal; the increase was mainly associated with warfarin, not non–vitamin K antagonist OACs.

**Meaning:**

This study suggests that more specific programs educating physicians and patients are needed to ensure that OACs, especially non–vitamin K OACs, are prescribed to eligible patients.

## Teaser

This quality improvement study assesses adherence to guideline-recommended oral anticoagulants, the temporal improvement in adherence, and the risk factors associated with oral anticoagulant prescriptions.

## Introduction

Atrial fibrillation (AF) is a known cardiac risk factor for ischemic stroke.^1,2,3^ Findings from contemporary registry-based observational studies from various geographical regions have consistently shown that patients with ischemic stroke and AF have worse prognoses, higher rates of medical and neurologic complications, and higher case fatality rates than those without AF.^4,5,6,7^ Nevertheless, stroke associated with AF is mostly preventable, and the use of oral anticoagulants (OACs), including warfarin and non–vitamin K oral anticoagulants (NOACs), was associated with effectively reducing ischemic stroke and systemic thromboembolism in high-risk patients with AF.^8,9,10^ However, the prescribing of OACs has not been well characterized for patients in China with ischemic stroke and AF, as previous studies have been limited in terms of sample size, geographical regions studied, or types of OACs prescribed.^11,12,13,14^

The Chinese Stroke Center Alliance (CSCA) is a national stroke care quality assessment and improvement program with the primary goal of improving the quality of care and outcomes for patients with stroke.^15^ Using the nationwide and rigorously assessed data on consecutively recruited patients from CSCA, we aimed to assess adherence to guideline-directed prescribing of OAC therapy for stroke prevention. We hypothesized that adherence to prescribing of OACs per guideline recommendations for patients with AF would improve over time.

## Methods

The data that support the findings from this study are available from the corresponding author upon reasonable request and after clearance by the ethics committee. This report follows the revised Standards for Quality Improvement Reporting Excellence (SQUIRE) reporting guideline for quality improvement studies.

### CSCA Program

The CSCA program is a national, hospital-based, multicenter, multifaceted quality improvement initiative for stroke and transient ischemic attack. This program was made available to all secondary and tertiary hospitals in China. The ethics committee of Beijing Tiantan Hospital approved the CSCA program. Each participating hospital received institutional review board approval to enroll participants without individual patient consent under a waiver of authorization and exemption because only deidentified data were collected from routine clinical practice. The protocol for case identification and data collection has been reported in detail elsewhere.^15^

Patients were enrolled if they had a primary diagnosis of stroke confirmed by brain computed tomography or magnetic resonance imaging. Data were collected by trained hospital personnel using the web-based Patient Management Tool (Medicine Innovation Research Center). The China National Clinical Research Center for Neurological Diseases served as the data analysis center and had an agreement to analyze the aggregate deidentified data for feedback on quality of care and for research purposes.

### Multifaceted Quality Improvement Initiative

The multifaceted quality improvement initiative included 2 parts, which were developed using the Plan-Do-Check-Act cycle. The first part consists of a web-based data collection and feedback system,^16^ which collects concurrent data via predefined logic features, range checks, and user alerts; provides feedback on key performance measures that were proven to be effective in a randomized clinical trial of multifaceted quality improvement intervention^17^; and allows participating hospitals to compare their performance with the past and current standards of other hospitals. The second part is the construction of stroke centers by collaborative workshops and webinars. More details can be found in the protocol of the CSCA.^15^

### Study Population

The CSCA enrolled 70 365 patients with ischemic stroke and AF from 1430 participating hospitals from August 1, 2015, to July 31, 2019. The presence of AF was ascertained by medical history taking, in-hospital diagnosis by electrocardiography, or 24-hour Holter monitoring. A total of 4319 patients were excluded owing to discharge against medical advice, missing discharge information, or transfer to a tertiary hospital.

For the remaining 66 046 patients with ischemic stroke and AF, we created 2 study populations for at-admission analysis and at-discharge analysis. We included 35 767 participants for the at-admission analysis after excluding those with AF diagnosed at discharge (n = 23 949), a CHA_2_DS_2_-VASc (cardiac failure or dysfunction, hypertension, age 65-74 [1 point] or ≥75 years [2 points], diabetes mellitus, and stroke, transient ischemic attack or thromboembolism [2 points]–vascular disease, and sex category [female]) score less than 2 (n = 4241), and missing OAC information prior to admission (n = 2089). For the at-discharge analysis, we included 49 531 patients after excluding in-hospital deaths (n = 1297), those with contraindications to OACs (n = 8689), those with a prescription for heparin or low-molecular-weight heparin (n = 3886), those missing information on OACs at discharge (n = 1935), and those with a prescription for 2 or more OACs at discharge (n = 708). A flowchart of study population identification is presented in eFigure 1 in the Supplement.

### Study Variables and Definitions

The OACs in this study included warfarin and NOACs; the latter included dabigatran, rivaroxaban, apixaban, and edoxaban. Adherence to guideline-recommended prescribing of OACs was assessed based on clinical management guidelines for patients with ischemic stroke from the Chinese Stroke Association or the American Heart Association/American Stroke Association.^18,19^ We collected data on sociodemographic characteristics, including age, sex, educational level, insurance type (urban employee basic medical insurance, urban resident basic medical insurance, new rural cooperative medical scheme, self-pay, and others),^20^ and per capita family income (calculated by dividing total family income by the number of family members). Data were also collected on whether patients had a history of AF, stroke and transient ischemic attack, carotid stenosis, coronary heart disease, myocardial infarction, hypertension, diabetes, dyslipidemia, or peripheral vascular disease. Information on medication use in the 6 months prior to stroke hospitalization was also collected. Stroke severity was measured by the National Institutes of Health Stroke Scale score (range, 0-42, with a higher score indicating greater stroke severity), with moderate or severe stroke defined by a score of 16 or more.^21,22^ Geographical regions were classified into eastern, central, and western, according to the *China Health and Family Planning Statistics Yearbook 2017*.^23^ Additional information on data collection and variable definition were described in the CSCA protocol.^15^

### Statistical Analysis

Patients for at-admission analysis were described overall and by OAC use on admission using counts with percentages for categorical variables and mean (SD) values or median values with interquartile ranges for continuous variables. Given the large sample size, comparisons between groups using traditional methods such as the Pearson χ^2^ test, independent *t* test, or Wilcoxon rank sum tests would have been susceptible to false-positive findings. Therefore, we used the absolute standardized difference (ASD) to compare patient characteristics between groups, with an ASD greater than 10% considered clinically significant.^24^ Cochran-Armitage trend tests were used to assess temporal trends of OAC use and contraindication to OACs from the third quarter of 2015 to the third quarter of 2019.

All *P* values were 2-sided, with *P* < .05 considered statistically significant. All statistical analyses were performed using SAS, version 9.4 (SAS Institute Inc). An SAS macro called %ggBaseline was used to analyze and report baseline characteristics automatically.^25^

## Results

### Baseline Characteristics

Among 35 767 patients with ischemic stroke and previous AF on admission, the mean (SD) age was 75.5 (9.2) years, and 18 785 patients (52.5%) were female. The median CHA_2_DS_2_-VASc score was 4.0 (interquartile range, 3.0-5.0).

### OAC Use Prior to Stroke Hospitalization

**Figure 1.**
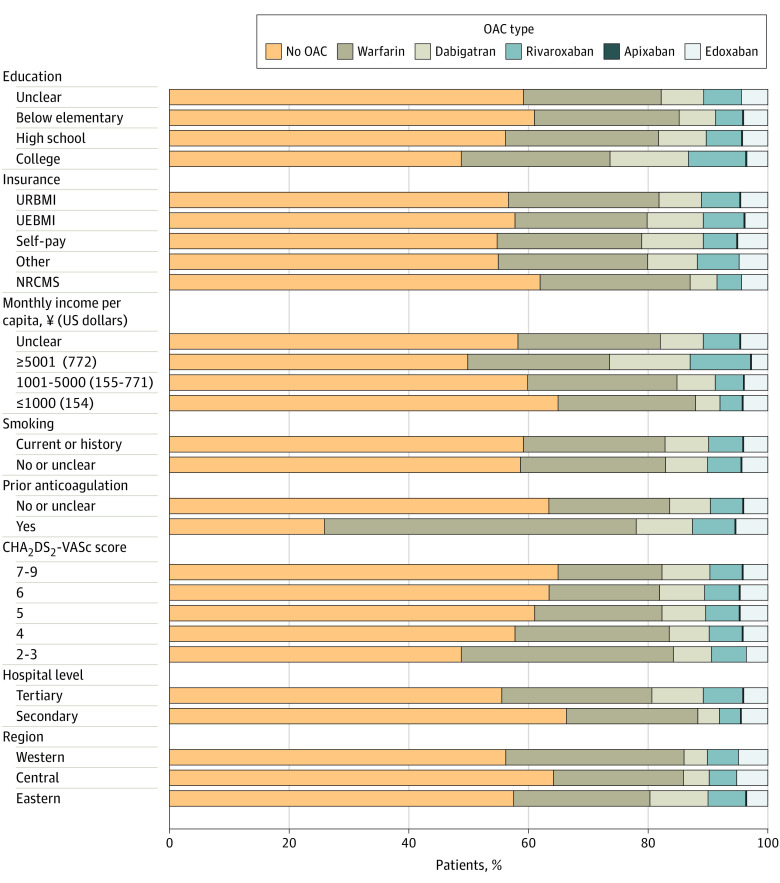
Patterns of Oral Anticoagulant (OAC) Prescription at Discharge in Selected Subgroups of Eligible Patients CHA_2_DS_2_-VASc indicates cardiac failure or dysfunction, hypertension, age 65-74 (1 point) or ≥75 years (2 points), diabetes, and stroke, transient ischemic attack or thromboembolism (2 points)–vascular disease, and sex category (female); NRCMS, new rural cooperative medical scheme; UEBMI, urban employee basic medical insurance; and URBMI, urban resident basic medical insurance.

At admission, 6303 of 35 767 (17.6%) patients with ischemic stroke and previous AF and with a CHA_2_DS_2_-VASc score of 2 or greater were taking OACs. The rate of OAC use prior to stroke hospitalization was 14.3% (20 of 140) in the third quarter of 2015 and increased to 21.1% (118 of 560) in the third quarter of 2019 (*P* < .001 for trend) (Figure 1). Compared with patients not taking OACs (n = 29 464), those taking OACs prior to stroke hospitalization were younger (mean [SD] age, 73.0 [10.0] vs 76.0 [8.9] years; ASD = 31.7%), had higher levels of education (high school, 1732 [27.5%] vs 6797 [23.1%]; ASD = 10.1%), were less likely to be covered by the new rural cooperative medical scheme (1956 [31.0%] vs 10 894 [37.0%]; ASD = 12.7%), and were more likely to be admitted to a tertiary hospital (4585 [72.7] vs 19 837 [67.3%]; ASD = 11.8%); a higher proportion had experienced prior stroke and transient ischemic attack (4061 [64.4%] vs 12 835 [43.6%]; ASD = 42.7%), carotid stenosis (225 [3.6%] vs 517 [1.8%]; ASD = 11.1%), coronary heart disease or myocardial infarction (1848 [29.3%] vs 6915 [23.5%]; ASD = 13.2%), dyslipidemia (1024 [16.2%] vs 2644 [9.0%]; ASD = 21.8%), and peripheral vascular disease (490 [7.8%] vs 1084 [3.7%]; ASD = 17.7%); and a higher proportion had a history of taking antiplatelet agents (2815 [44.7%] vs 9909 [33.6%]; ASD = 22.9%), antihypertensive agents (3811 [60.5%] vs 16 126 [54.7%]; ASD = 11.8%), glucose-lowering medications (1309 [20.8%] vs 4661 [15.8%]; ASD = 13.0%), and lipid-lowering medications (3123 [49.5%] vs 6202 [21.0%]; ASD = 62.5%) (Table 1).

**Table 1.** Baseline Characteristics of Patients With Prior AF and a CHA_2_DS_2_-VASc Score of 2 or Higher

Characteristic	Patients, No. (%)			ASD, %
	Total (N = 35 767)	OAC (n = 6303)	No OAC (n = 29 464)	
Age, mean (SD), y	75.5 (9.2)	73.0 (10.0)	76.0 (8.9)	31.7
Male	16 982 (47.5)	3102 (49.2)	13 880 (47.1)	4.2
Female	18 785 (52.5)	3201 (50.8)	15 584 (52.9)	4.2
Educational level				
College	977 (2.7)	231 (3.7)	746 (2.5)	6.9
High school	8529 (23.8)	1732 (27.5)	6797 (23.1)	10.1
Below elementary school	13 465 (37.6)	2020 (32.0)	11 445 (38.8)	14.3
Unclear	12 796 (35.8)	2320 (36.8)	10 476 (35.6)	2.5
Insurance				
UEBMI	12 420 (34.7)	2333 (37.0)	10 087 (34.2)	5.9
URBMI	7249 (20.3)	1312 (20.8)	5937 (20.2)	1.5
NRCMS	12 850 (35.9)	1956 (31.0)	10 894 (37.0)	12.7
Self-pay	1703 (4.8)	343 (5.4)	1360 (4.6)	3.7
Other	1545 (4.3)	359 (5.7)	1186 (4.0)	7.9
Monthly income per capita, ¥ (US dollars)^a^				
≤1000 (154)	2568 (7.2)	433 (6.9)	2135 (7.2)	1.2
1001-5000 (155-771)	12 497 (34.9)	2140 (34.0)	10 357 (35.2)	2.5
≥5001 (772)	2155 (6.0)	464 (7.4)	1691 (5.7)	6.9
Unclear	18 547 (51.9)	3266 (51.8)	15 281 (51.9)	0.2
Current smoker or history of smoking	8874 (24.8)	1566 (24.8)	7308 (24.8)	0.0
Drinking	5545 (15.5)	1047 (16.6)	4498 (15.3)	3.6
Risk factors known before admission				
Prior stroke or TIA	16 896 (47.2)	4061 (64.4)	12 835 (43.6)	42.7
CHD or MI	8763 (24.5)	1848 (29.3)	6915 (23.5)	13.2
Hypertension	24 852 (69.5)	4193 (66.5)	20 659 (70.1)	7.7
Diabetes	7460 (20.9)	1467 (23.3)	5993 (20.3)	7.3
Dyslipidemia	3668 (10.3)	1024 (16.2)	2644 (9.0)	21.8
Heart failure	3274 (9.2)	672 (10.7)	2602 (8.8)	6.4
Carotid stenosis	742 (2.1)	225 (3.6)	517 (1.8)	11.1
PVD	1574 (4.4)	490 (7.8)	1084 (3.7)	17.7
Medication before admission				
Antiplatelet medication	12 724 (35.6)	2815 (44.7)	9909 (33.6)	22.9
Antihypertension medication	19 937 (55.7)	3811 (60.5)	16 126 (54.7)	11.8
Glucose-lowering medication	5970 (16.7)	1309 (20.8)	4661 (15.8)	13.0
Lipid-lowering medication	9325 (26.1)	3123 (49.5)	6202 (21.0)	62.5
CHA_2_DS_2_-VASc score, median (IQR)	4.0 (3.0-5.0)	4.0 (3.0-5.0)	4.0 (3.0-5.0)	
Hospital level				
Secondary	11 345 (31.7)	1718 (27.3)	9627 (32.7)	11.8
Tertiary	24 422 (68.3)	4585 (72.7)	19 837 (67.3)	11.8
Region				
Eastern	19 589 (54.8)	3341 (53.0)	16 248 (55.1)	4.2
Central	8982 (25.1)	1504 (23.9)	7478 (25.4)	3.5
Western	7196 (20.1)	1458 (23.1)	5738 (19.5)	8.8

Abbreviations: AF, atrial fibrillation; ASD, absolute standardized difference; CHA_2_DS_2_-VASc, cardiac failure or dysfunction, hypertension, age 65-74 (1 point) or ≥75 years (2 points), diabetes, and stroke, transient ischemic attack or thromboembolism (2 points)–vascular disease, and sex category (female); CHD, coronary heart disease; IQR, interquartile range; MI, myocardial infarction; NRCMS, new rural cooperative medical scheme; OAC, oral anticoagulation; PVD, peripheral vascular disease; TIA, transient ischemic attack; UEBMI, urban employee basic medical insurance; URBMI, urban resident basic medical insurance.

^a^ Conversion rate, $1 = ¥6.41.

### Contraindications to OACs at Discharge

Among 66 046 patients with ischemic stroke and AF, 1297 in-hospital deaths were excluded and 64 749 patients were analyzed for contraindications to OACs. A total of 8689 patients (13.4%) had at least 1 contraindication to OACs, and the 3 most common contraindications were bleeding risk (5452 [8.4%]), family member or patient preference (2943 [4.5%]), and terminal illness (443 [0.7%]) (Table 2). The rate of contraindications decreased from 16.7% (329 of 1973) in 2015 to 12.7% (1487 of 11 749) in 2019 (24% relative reduction; *P* < .001 for trend). Bleeding risk as a contraindication decreased over time from 11.1% (219 of 1973) in 2015 to 8.1% (949 of 11 749) in 2019 (27% relative reduction; *P* = .001 for trend), and family member or patient preference as a contraindication also decreased over time, from 5.0% (99 of 1973) in 2015 to 4.2% (494 of 11 749) in 2019 (16% relative reduction; *P* < .001 for trend).

**Table 2.** Contraindications to OACs Among Discharged Patients With a CHA_2_DS_2_-VASc Score of 2 or Higher

OAC contraindication	Patients, No. (%)						*P* value for trend
	Total (N = 64 749)	2015 (n = 1973)	2016 (n = 13 857)	2017 (n = 16 391)	2018 (n = 20 779)	2019 (n = 11 749)	
Contraindication	8689 (13.4)	329 (16.7)	2044 (14.8)	2247(13.7)	2582 (12.4)	1487 (12.7)	<.001
Bleeding risk	5452 (8.4)	219 (11.1)	1185 (8.6)	1402 (8.6)	1697 (8.2)	949 (8.1)	.001
Patient refusal	2943 (4.5)	99 (5.0)	755 (5.4)	767 (4.7)	828 (4.0)	494 (4.2)	<.001
Allergy or comorbid illness	300 (0.5)	7 (0.4)	90 (0.6)	73 (0.4)	83 (0.4)	47 (0.4)	.009
Falls	176 (0.3)	4 (0.2)	49 (0.4)	46 (0.3)	48 (0.2)	29 (0.2)	.12
Mental health	103 (0.2)	4 (0.2)	26 (0.2)	26 (0.2)	34 (0.2)	13 (0.1)	.15
Severe adverse effect	145 (0.2)	5 (0.3)	32 (0.2)	38 (0.2)	47 (0.2)	23 (0.2)	.53
Terminal illness	443 (0.7)	14 (0.7)	102 (0.7)	108 (0.7)	134 (0.6)	85 (0.7)	.76

Abbreviations: CHA_2_DS_2_-VASc, cardiac failure or dysfunction, hypertension, age 65-74 (1 point) or ≥75 years (2 points), diabetes, and stroke, transient ischemic attack or thromboembolism (2 points)–vascular disease, and sex category (female); OAC, oral anticoagulant.

### OAC Prescription at Discharge

**Figure 2.**
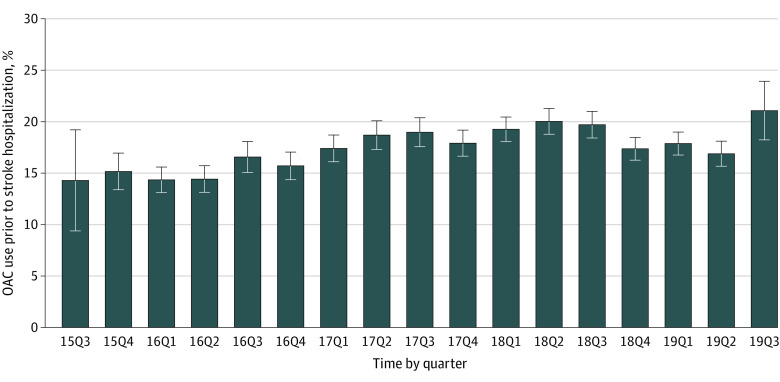
Trends of Oral Anticoagulant (OAC) Use Prior to Stroke Hospitalization *P* < .001 for trend. Error bars indicate 95% CIs. 15Q3 to 19Q3 indicate the third quarter of 2015 to the third quarter of 2019.

At discharge, 20 390 of 49 531 eligible patients (41.2%) without contraindications and with a CHA_2_DS_2_-VASc score of 2 or more were prescribed an OAC. The most commonly prescribed OAC was warfarin (11 956 [24.2%]), followed by dabigatran (3514 [7.1%]), rivaroxaban (2796 [5.6%]), and edoxaban (2091 [4.2%]). Detailed OAC prescription patterns are shown in Figure 2. Patients with a college education, higher income, and prior use of anticoagulants, as well as those admitted to tertiary hospitals, were more likely to be prescribed OACs. Higher CHA_2_DS_2_-VASc scores were not associated with a higher rate of OAC prescription.

**Figure 3.**
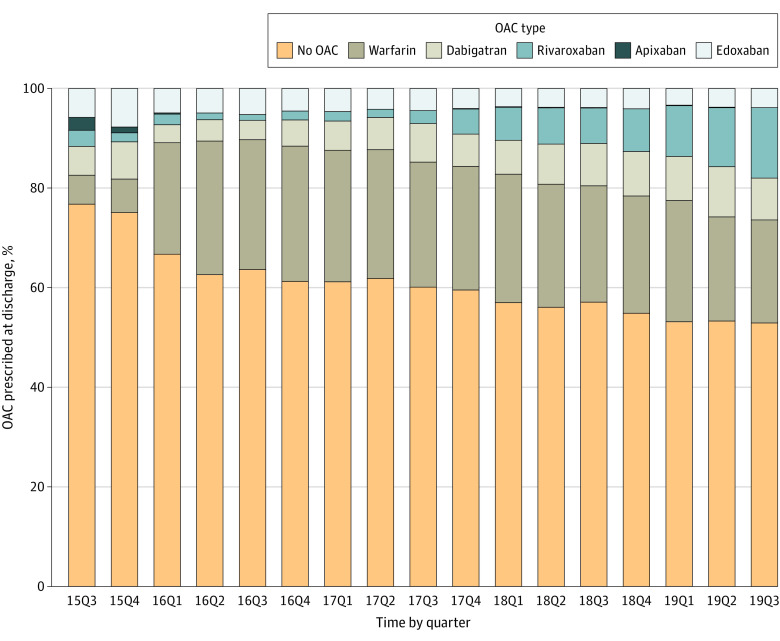
Trends of Oral Anticoagulant (OAC) Prescription at Discharge Among Eligible Patients 15Q3 to 19Q3 indicate the third quarter of 2015 to the third quarter of 2019.

To assess OAC prescription changes at discharge from 2015 to 2019, we assessed the quarterly temporal trend (Figure 3). Oral anticoagulant prescriptions at discharge increased from 23.2% (36 of 155) in the third quarter of 2015 to 47.1% (403 of 856) in the third quarter of 2019 (*P* < .001 for trend). The largest increase was observed for warfarin (from 5.8% [9 of 155] to 20.7% [177 of 856]), followed by rivaroxaban (from 3.2% [5 of 155] to 14.1% [121 of 856]) and dabigatran (from 5.8% [9 of 155] to 8.4% [72 of 856]).

In addition, we assessed the variation in OAC prescriptions across 604 hospitals with at least 100 patients. The rates of OAC prescription at hospitals ranged from 0% to 95%, with a median of 38.8% (interquartile range, 23.1%-52.9%) (eFigure 2 in the Supplement).

### Factors Associated With OAC Prescription at Discharge

Patients prescribed OACs at discharge (n = 20 390) were younger than those not prescribed OACs at discharge (n = 29 141) (mean [SD] age, 71.7 [10.6] vs 74.6 [10.2] years; ASD = 27.9%), less likely to be covered by the new rural cooperative medical scheme (7013 [34.4%] vs 11 443 [39.3%]; ASD = 10.2%), had a lower rate of hypertension (11 919 [58.5%] vs 18 633 [63.9%]; ASD = 11.1%), had a lower rate of prior antiplatelet agent use (4616 [22.6%] vs 8063 [27.7%]; ASD = 11.8%), had a higher rate of prior anticoagulant agent use (4483 [22.0%] vs 1563 [5.4%]; ASD = 49.7%), were more likely to be from tertiary hospitals (15 373 [75.4%] vs 19 247 [66.0%]; ASD = 20.8%), and were less likely to be from hospitals located in the central region of China (4266 [20.9%] vs 7652 [26.3%]; ASD = 12.7%) (eTable 1 in the Supplement). Multivariable logistic models showed that older age (adjusted odds ratio [aOR] per 5 year increase, 0.89; 95% CI, 0.89-0.90), lower levels of education (aOR for below elementary school, 0.84; 95% CI, 0.74-0.95), being covered by the new rural cooperative medical scheme (aOR, 0.92; 95% CI, 0.87-0.96), lower levels of family income (aOR for ≤¥1000 [$154], 0.66; 95% CI, 0.59-0.73), having certain comorbid conditions (including stroke or transient ischemic attack [aOR, 0.78; 95% CI, 0.75-0.82], hypertension [aOR, 0.84; 95% CI, 0.80-0.89], diabetes [aOR, 0.91; 95% CI, 0.83-0.99], dyslipidemia [aOR, 0.87; 95% CI, 0.80-0.94], carotid stenosis [aOR, 0.83; 95% CI, 0.69-0.98], and peripheral vascular disease [aOR, 0.80; 95% CI, 0.71-0.90]), having had a previous prescription of antiplatelet agents (aOR, 0.70; 95% CI, 0.66-0.74), and being from a secondary hospital (aOR, 0.71; 95% CI, 0.68-0.74) or a hospitals located in the central region of China (aOR, 0.80; 95% CI, 0.75-0.84) were associated with a lower rate of OAC prescription at discharge. Conversely, previous AF diagnosis (aOR, 1.08; 95% CI, 1.04-1.13) as well as prior use of anticoagulant agents (aOR, 5.17; 95% CI, 4.84-5.52), antihypertensive agents (aOR,1.08; 95% CI, 1.02-1.14), and lipid-lowering medications (aOR, 1.10; 95% CI, 1.03-1.17) were associated with higher odds of OAC prescription at discharge (eTable 2 in the Supplement).

## Discussion

In this multicenter quality improvement study, we used data on patients with ischemic stroke and prior AF in China to assess adherence to guideline-recommended prescribing of OAC therapy at admission and OAC prescription at discharge. Our analysis yielded several findings. First, adherence to guidelines for prescribing OAC therapy improved over time but remains low, with warfarin as the most commonly prescribed OAC and the fastest growing in use. Furthermore, bleeding risk was the most common concern among documented contraindications to OACs. Finally, we identified risk factors associated with being less likely to be prescribed an OAC at discharge.

Atrial fibrillation is a well-recognized risk factor for stroke, and previous studies from both randomized clinical trials and registry studies showed that OAC use prior to admission was associated with more favorable outcomes.^10,26,27^ However, OACs, particularly NOACs, are underprescribed in China. Data from the China National Stroke Registry showed that only 21% of patients with acute ischemic stroke and AF were prescribed anticoagulants at discharge from 2012 to 2013^28^ compared with more than 90% in the Get With the Guidelines–Stroke program in the United States from 2003 to 2009.^29,30^ The community-based data from the China National Stroke Screening Survey during 2013 and 2014 showed that, among patients with ischemic stroke and AF, only 2.2% were taking OACs, and of them, 98.2% were taking warfarin.^13^ Another community-based survey of 47 841 adults aged 45 years or older in 7 geographical regions of China between 2014 and 2016 showed that AF prevalence was 1.8% (95% CI, 1.7%-1.9%), with an estimation of 7.9 million patients (95% CI, 7.4 million to 8.4 million patients) with AF in China. However, only 6.0% of patients with high-risk AF received anticoagulation therapy.^31^ Therefore, more efforts at the community level are needed for stroke prevention. Considerable efforts were made to improve adherence to evidence-based performance measures among patients with acute ischemic stroke in China. A multifaceted quality improvement intervention improved the proportion of patients with ischemic stroke and AF receiving anticoagulation to 40.6%.^17^ In the present analysis, the proportion of eligible patients receiving anticoagulation was 47.1% in 2019, thus showing further improvement. However, large gaps were still present compared with the rates observed in developed countries.^29,30^

The increase in OAC prescriptions was mainly associated with warfarin, not NOACs. NOACs offer a more favorable risk-benefit profile, with significant reductions in intracranial hemorrhage.^10,32^ Although the medical community in China has been making efforts to increase OAC and NOAC prescriptions for patients with AF, the shift from antiplatelet agents to anticoagulant agents remains slow, according to the Chinese Stroke Association guidelines for the clinical management of cerebrovascular disorders.^18^ Higher cost and lack of insurance coverage for NOACs for most Chinese patients prohibited the use of NOACs. To provide financial resources, the Ministry of Human Resources and Social Security of the People’s Republic of China released the 2017 Medicine Catalogue for National Basic Medical Insurance, Injury Insurance, and Maternity Insurance in February 2017. This initiative covered the fees for dabigatran and rivaroxaban for patients with AF. Rivaroxaban prescriptions subsequently increased from 3.2% (5 of 155) in the first quarter of 2017 to 14.1% (121 of 856) in the third quarter of 2019.

Although we observed improvements, we also found wide variation in the rates of OAC prescription among hospitals, ranging from 0% to 95%. Future studies should focus on improving the rates of OAC prescription for hospitals at the lower end of the distribution. Because one of the most common reasons for not prescribing OACs is concern about hemorrhage, patient and family education on the benefits and risks associated with using OACs should be made available and widely adopted by health care professionals.

### Limitations

This study has several limitations. First, this analysis was based on the voluntary enrollment of participating hospitals and does not have an elaborately designed sampling frame, which may have biased our study to include hospitals with better stroke care than the national average. Second, data on detailed OAC types and the quality control of OAC use prior to stroke hospitalization were not collected; therefore, the percentages of different OAC types and of patients with well-controlled anticoagulation therapy could not be calculated. Third, postdischarge OAC compliance and clinical outcomes were not assessed because no follow-up data were collected. Fourth, the results of this study may not be generalizable to other countries because participants were restricted to Chinese patients, and many of the issues studied are specific to the Chinese context. Despite these limitations, the present analysis provides a novel contribution by characterizing OAC prescriptions among patients with ischemic stroke and AF, providing insights into stroke prevention and control at a national level.

## Conclusions

Although adherence to guideline-recommended prescribing of OAC therapy to eligible patients with indications of ischemic stroke and AF improved over time, the prescription rate remains suboptimal. Programs and interventions to increase the rate of prescription for OACs, in particular NOACs, are needed to improve the quality of care, particularly for vulnerable subgroups by age, socioeconomic status, and presence of comorbid conditions.

## Notes

Supplement. **eFigure 1.** Flowchart of Study Population Identification **eFigure 2.** Variation in OAC Prescription at Discharge **eTable 1.** Baseline Characteristics by OAC Prescription at Discharge **eTable 2.** Factors Associated With OAC Prescription at Discharge
