# Errors in Labeling Figures 1 and 2

**DOI:** 10.1001/jamanetworkopen.2022.19838

**Published:** 2022-06-13

In the Original Investigation titled “Assessment of Trends in Guideline-Based Oral Anticoagulant Prescription for Patients With Ischemic Stroke and Atrial Fibrillation in China,”^1^ published July 29, 2021, Figures 1 and 2 were mislabeled. The labels “Figure 1” and “Figure 2” should be switched to be consistent with the citations in the main text. This article has been corrected.^1^
