# Supplementary material for: Assessment of Trends in Guideline-Based Oral Anticoagulant Prescription for Patients With Ischemic Stroke and Atrial Fibrillation in China
Source: JAMA Netw Open. 2021 Jul 29;4(7):e2118816. doi: 10.1001/jamanetworkopen.2021.18816 (PMC8322995; doi:10.1001/jamanetworkopen.2021.18816)
Supplement: Supplement. — eFigure 1. Flowchart of Study Population Identification eFigure 2. Variation in OAC Prescription at Discharge eTable 1. Baseline Characteristics by OAC Prescription at Discharge eTable 2. Factors Associated With OAC Prescription at Discharge [file jamanetwopen-e2118816-s001.pdf]

Supplementary Online Content

Gu HQ, Yang X, Wang CJ, et al. Assessment of trends in guideline-based oral anticoagulant prescription for patients with ischemic stroke and atrial fibrillation in China. *JAMA Netw Open.* 2021;4(7):e2118816. doi:10.1001/jamanetworkopen.2021.18816

eFigure 1. Flowchart of Study Population Identification
eFigure 2. Variation in OAC Prescription at Discharge
eTable 1. Baseline Characteristics by OAC Prescription at Discharge
eTable 2. Factors Associated With OAC Prescription at Discharge

This supplementary material has been provided by the authors to give readers additional information about their work.

© 2021 Gu HQ et al. *JAMA Network Open.*

eFigure 1. Flowchart of Study Population Identification

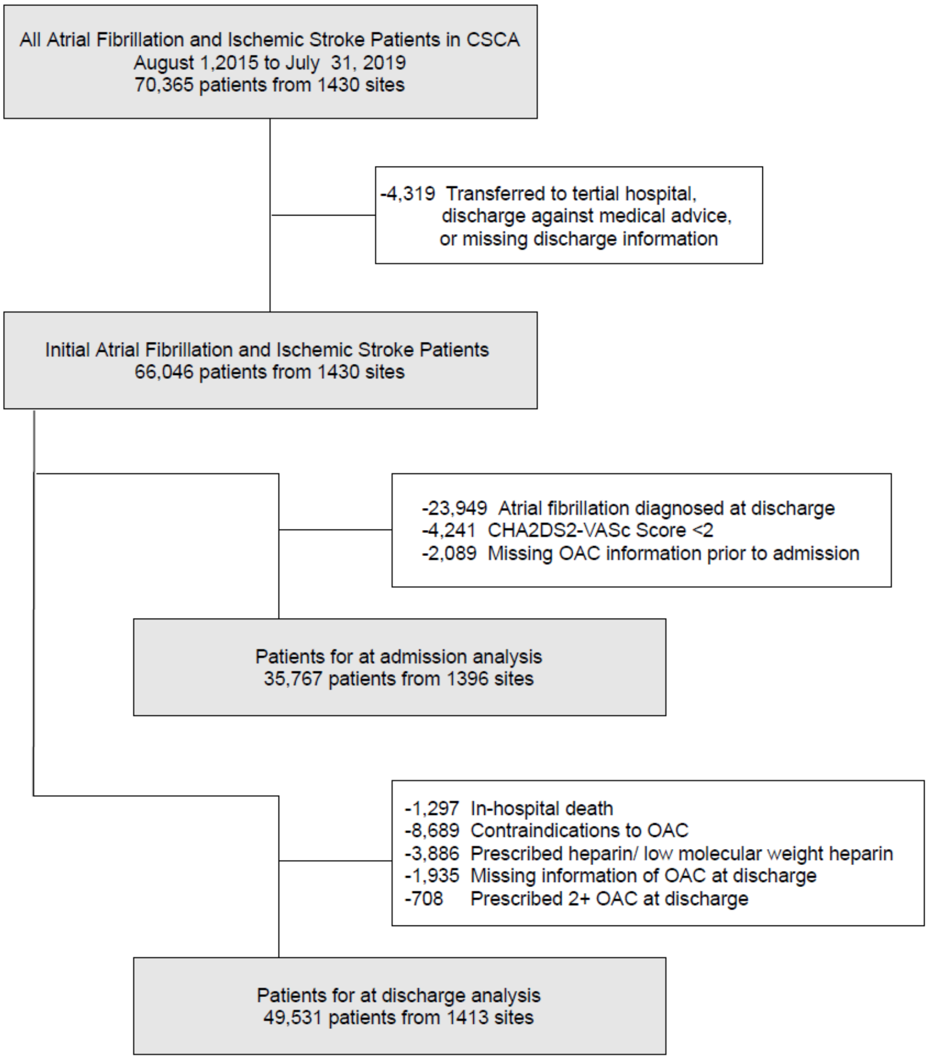

Abbreviations: CSCA, China Stroke Center Alliance; CHA_2DS_2-VASc, cardiac failure or dysfunction, hypertension, age 65-74 (1 point) or ≥75 years (2 points), diabetes mellitus, and stroke, transient ischemic attack or thromboembolism (2 points)–vascular disease, and sex category (female); OAC, oral anticoagulation.

© 2021 Gu HQ et al. *JAMA Network Open.*

eFigure 2. Variation in OAC Prescription at Discharge

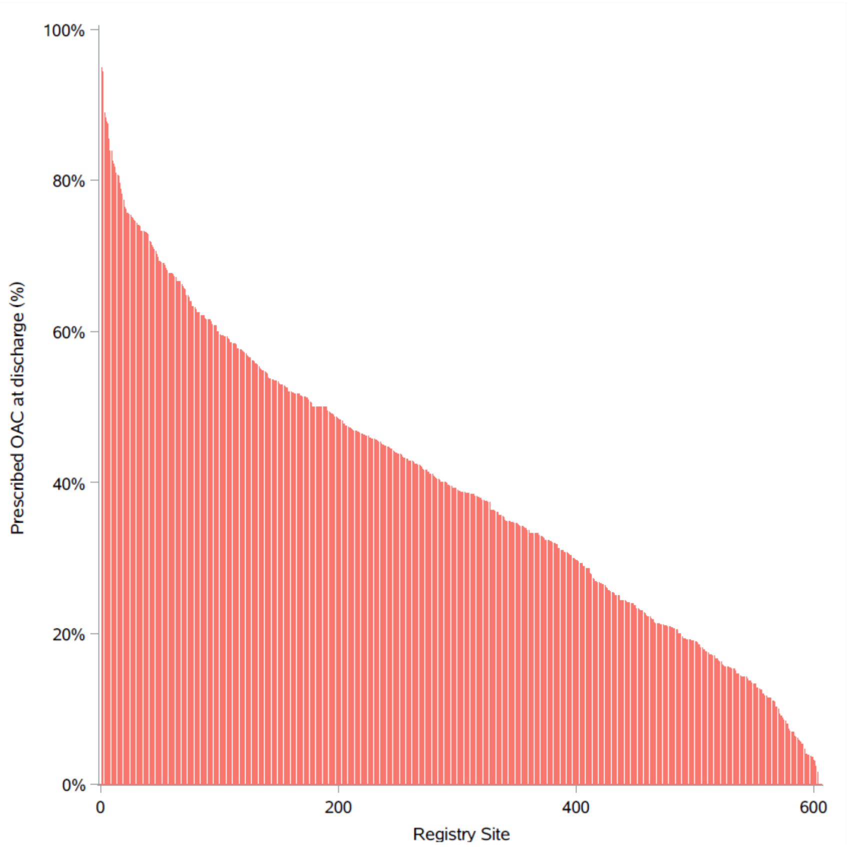

Abbreviations: OAC, oral anticoagulation.

© 2021 Gu HQ et al. *JAMA Network Open.*

eTable 1. Baseline Characteristics by OAC Prescription at Discharge

Variables	Total (N=49531)	On OAC (N=20390)	Not on OAC (N=29141)	ASD (%)
Age	73.4±10.4	71.7±10.6	74.6±10.2	27.9
Male	26028 (52.5)	10749 (52.7)	15279 (52.4)	0.6
Female	23503 (47.5)	9641 (47.3)	13862 (47.6)	0.6
Educational level				
College	1249 (2.5)	640 (3.1)	609 (2.1)	6.3
High school	11889 (24.0)	5210 (25.6)	6679 (22.9)	6.3
Below elementary school	18274 (36.9)	7122 (34.9)	11152 (38.3)	7.1
Unclear	18119 (36.6)	7418 (36.4)	10701 (36.7)	0.6
Insurance				
UEBMI	15464 (31.2)	6529 (32.0)	8935 (30.7)	2.8
URBMI	10529 (21.3)	4556 (22.3)	5973 (20.5)	4.4
NRCMS	18456 (37.3)	7013 (34.4)	11443 (39.3)	10.2
Self-pay	2862 (5.8)	1294 (6.3)	1568 (5.4)	3.8
Other	2220 (4.5)	998 (4.9)	1222 (4.2)	3.4
Monthly income per capita, ¥ (US dollars)[a]				
≤1000 (154)	3482 (7.0)	1220 (6.0)	2262 (7.8)	7.1
1001-5000 (155-771)	16716 (33.7)	6697 (32.8)	10019 (34.4)	3.4
≥5001 (772)	2760 (5.6)	1383 (6.8)	1377 (4.7)	9.0
Unclear	26573 (53.6)	11090 (54.4)	15483 (53.1)	2.6
Current smoker or history of smoking	13620 (27.5)	5563 (27.3)	8057 (27.6)	0.7
Drinking	8730 (17.6)	3609 (17.7)	5121 (17.6)	0.3
Risk factors known before admission				
Prior Stroke or TIA	17279 (34.9)	6598 (32.4)	10681 (36.7)	9.1
CHD or MI	10550 (21.3)	4425 (21.7)	6125 (21.0)	1.7
Hypertension	30552 (61.7)	11919 (58.5)	18633 (63.9)	11.1
Diabetes	8475 (17.1)	3333 (16.3)	5142 (17.6)	3.5
Dyslipidemia	3419 (6.9)	1322 (6.5)	2097 (7.2)	2.8
Heart failure	2715 (5.5)	1071 (5.3)	1644 (5.6)	1.3
Carotid stenosis	656 (1.3)	238 (1.2)	418 (1.4)	1.8
PVD	1387 (2.8)	506 (2.5)	881 (3.0)	3.1
Atrial fibrillation	31232 (63.1)	13305 (65.3)	17927 (61.5)	7.9
Medication before admission				
Antiplatelet medication	12679 (25.6)	4616 (22.6)	8063 (27.7)	11.8

© 2021 Gu HQ et al. *JAMA Network Open.*

Anticoagulants	6046 (12.2)	4483 (22.0)	1563 (5.4)	49.7
Antihypertension medication	23314 (47.1)	9307 (45.6)	14007 (48.1)	5.0
Glucose-lowering medication	6581 (13.3)	2630 (12.9)	3951 (13.6)	2.1
Lipid-lowering medication	8987 (18.1)	3806 (18.7)	5181 (17.8)	2.3
CHA_2DS_2-VASc, median (IQR)	3.0 (2.0–5.0)	3.0 (2.0–4.0)	3.0 (2.0–5.0)	
Hospital level				
Secondary	14911 (30.1)	5017 (24.6)	9894 (34.0)	20.8
Tertiary	34620 (69.9)	15373 (75.4)	19247 (66.0)	20.8
Region				
Eastern	26270 (53.0)	11160 (54.7)	15110 (51.9)	5.6
Central	11918 (24.1)	4266 (20.9)	7652 (26.3)	12.7
Western	11343 (22.9)	4964 (24.3)	6379 (21.9)	5.7

Abbreviations: AF, atrial fibrillation; ASD, absolute standardized difference; CHD, coronary heart disease; IQR, interquartile range; MI, myocardial infarction; NRCMS, new rural cooperative medical scheme; OAC, oral anticoagulation; PVD, peripheral vascular disease; TIA, transient ischemic attack; UEBMI, urban employee basic medical insurance; URBMI, urban resident basic medical insurance.

[a]Conversion rate, $1 = ¥6.41.

© 2021 Gu HQ et al. *JAMA Network Open.*

eTable 2. Factors Associated With OAC Prescription at Discharge

	Unadjusted Analysis		Adjusted Analysis	
Factor	OR (95% CI)	P Value	OR (95% CI)	P Value
Age per 5 y	0.88 (0.87–0.88)	<.0001	0.89 (0.89–0.90)	<.0001
Female	0.99 (0.95–1.02)	0.5308	1.02 (0.98–1.07)	0.3042
Educational level				
College	1.0 (Reference)		1.0 (Reference)	
Below elementary school	0.61 (0.54–0.68)	<.0001	0.84 (0.74–0.95)	0.0058
High school	0.74 (0.66–0.83)	<.0001	0.86 (0.76–0.97)	0.0186
Unclear	0.66 (0.59–0.74)	<.0001	0.81 (0.71–0.92)	0.0011
Insurance				
UEBMI	1.0 (Reference)		1.0 (Reference)	
URBMI	1.04 (0.99–1.10)	0.0928	1.07 (1.01–1.13)	0.0201
NRCMS	0.84 (0.80–0.88)	<.0001	0.92 (0.87–0.96)	0.0008
Self-pay	1.13 (1.04–1.22)	0.0030	0.95 (0.87–1.04)	0.2575
Other	1.12 (1.02–1.22)	0.0149	1.04 (0.95–1.15)	0.3893
Monthly income per capita, ¥ (US dollars)[a]				
≤1000 (154)	0.54 (0.48–0.59)	<.0001	0.66 (0.59–0.73)	<.0001
1001-5000 (155-771)	0.67 (0.61–0.72)	<.0001	0.71 (0.65–0.77)	<.0001
≥5001 (772)	1.0 (Reference)		1.0 (Reference)	
Unclear	0.71 (0.66–0.77)	<.0001	0.77 (0.71–0.84)	<.0001
Current smoker or history of smoking	0.98 (0.94–1.02)	0.3701	0.97 (0.92–1.02)	0.2056
Drinking	1.01 (0.96–1.06)	0.7158	0.96 (0.90–1.02)	0.1585
Risk factors known before admission				
Prior Stroke or TIA	0.83 (0.80–0.86)	<.0001	0.78 (0.75–0.82)	<.0001
CHD or MI	1.04 (1.00–1.09)	0.0676	0.98 (0.93–1.03)	0.4032
Hypertension	0.79 (0.76–0.82)	<.0001	0.84 (0.80–0.89)	<.0001
Diabetes mellitus	0.91 (0.87–0.96)	0.0002	0.91 (0.83–0.99)	0.0310
Dyslipidemia	0.89 (0.83–0.96)	0.0021	0.87 (0.80–0.94)	0.0006
Heart Failure	0.93 (0.86–1.00)	0.0613	0.96 (0.88–1.05)	0.4013
Carotid stenosis	0.81 (0.69–0.95)	0.0106	0.83 (0.69–0.98)	0.0328
PVD	0.82 (0.73–0.91)	0.0003	0.80 (0.71–0.90)	0.0003
Atrial fibrillation	1.17 (1.13–1.22)	<.0001	1.08 (1.04–1.13)	0.0001
Medication before admission				
Antiplatelet medication	0.76 (0.73–0.80)	<.0001	0.70 (0.66–0.74)	<.0001
Antihypertension	0.91 (0.88–0.94)	<.0001	1.08 (1.02–1.14)	0.0061

© 2021 Gu HQ et al. *JAMA Network Open.*

medication				
Glucose-lowering medication	0.94 (0.90–1.00)	0.0333	0.99 (0.90–1.09)	0.8427
Lipid-lowering medication	1.06 (1.01–1.11)	0.0117	1.10 (1.03–1.17)	0.0042
Anticoagulation	4.97 (4.68–5.28)	<.0001	5.17 (4.84–5.52)	<.0001
Hospital level				
Secondary	0.63 (0.61–0.66)	<.0001	0.71 (0.68–0.74)	<.0001
Tertiary	1.0 (Reference)		1.0 (Reference)	
Region				
Central	0.72 (0.68–0.76)	<.0001	0.80 (0.75–0.84)	<.0001
Eastern	0.95 (0.91–0.99)	0.0213	1.02 (0.97–1.07)	0.5183
Western	1.0 (Reference)		1.0 (Reference)	

Abbreviations: AF, atrial fibrillation; ASD, absolute standardized difference; CHD, coronary heart disease; CI, confidence interval; IQR, interquartile range; MI, myocardial infarction; NRCMS, new rural cooperative medical scheme; OR, odds ratio; PVD, peripheral vascular disease; TIA, transient ischemic attack; UEBMI, urban employee basic medical insurance; URBMI, urban resident basic medical insurance.

[a]Conversion rate, $1 = ¥6.41.

© 2021 Gu HQ et al. *JAMA Network Open.*
